# Bombyx mori Nucleopolyhedrovirus (BmNPV) Induces G2/M Arrest to Promote Viral Multiplication by Depleting *BmCDK1*

**DOI:** 10.3390/insects12121098

**Published:** 2021-12-08

**Authors:** Qin Xiao, Zhan-Qi Dong, Yan Zhu, Qian Zhang, Xiu Yang, Miao Xiao, Peng Chen, Cheng Lu, Min-Hui Pan

**Affiliations:** 1 State Key Laboratory of Silkworm Genome Biology, Southwest University, Chongqing 400716, China; XZ953179866@163.com (Q.X.); zqdong@swu.edu.cn (Z.-Q.D.); ZY1589230267@163.com (Y.Z.); qianzhang126@126.com (Q.Z.); pomeloyx@163.com (X.Y.); xm11250920@163.com (M.X.); pjchen@swu.edu.cn (P.C.); 2 Key Laboratory of Sericultural Biology and Genetic Breeding, Ministry of Agriculture and Rural Affairs, Southwest University, Chongqing 400716, China

**Keywords:** Bombyx mori, BmNPV, Cell cycle, *BmCDK1*, BmNPV *iap1*

## Abstract

**Simple Summary:**

Baculoviruses arrest the cell cycle in the S or G2/M phase in insect cells, but the exact mechanism of this process still remains obscure. Bombyx mori nucleopolyhedrovirus (BmNPV), one of the best characterized baculoviruses, is an important pathogen in silkworms. In the present study, we determined that downregulation of *BmCDK1* and *BmCyclin B* expression was required for BmNPV-mediated G2/M phase arrest, which plays an essential role in facilitating BmNPV replication. Further investigations showed that BmNPV IAP1 interacted with BmCDK1. The overexpression of the BmNPV *iap1* gene led to the accumulation of cells in the G2/M phase, and BmNPV *iap1* gene knockdown attenuated the effect of BmNPV-mediated G2/M phase arrest. These findings enhance the understanding of BmNPV pathogenesis, and indicate a novel mechanism through which baculoviruses impact the cell cycle progression.

**Abstract:**

Understanding virus–host interaction is very important for delineating the mechanism involved in viral replication and host resistance. Baculovirus, an insect virus, can cause S or G2/M phase arrest in insect cells. However, the roles and mechanism of Baculovirus-mediated S or G2/M phase arrest are not fully understood. Our results, obtained using flow cytometry (FCM), tubulin-labeling, BrdU-labeling, and CellTiter 96^®^ AQueous One Solution Cell Proliferation Assay (MTS), showed that Bombyx mori nucleopolyhedrovirus (BmNPV) induced G2/M phase arrest and inhibited cellular DNA replication as well as cell proliferation in BmN-SWU1 cells. We found that BmNPV induced G2/M arrest to support its replication and proliferation by reducing the expression of *BmCDK1* and *BmCyclin B*. Co-immunoprecipitation assays confirmed that BmNPV IAP1 interacted with BmCDK1. BmNPV *iap1* was involved in the process of BmNPV-induced G2/M arrest by reducing the content of BmCDK1. Taken together, our results improve the understanding of the virus–host interaction network, and provide a potential target gene that connects apoptosis and the cell cycle.

## 1. Introduction

Viruses are obligate intracellular parasites, and usually utilize proteins and enzymes encoded by host for their multiplication. With the co-evolution of viruses and hosts, viruses continually develop various strategies to utilize hosts’ components and escape hosts’ antiviral surveillance [1,2,3,4]. One prominent example is that viruses manipulate the cell cycle progression of their host cells. Generally, viruses delay, block, or prolong the G1 phase to prevent competition with the host cell for DNA replication materials [5,6,7], the S phase to utilize cellular DNA replication materials [8,9], or the G2/M phase to utilize the cellular cytoskeleton and escape from innate immune sensing [10,11,12].

The progression of cell cycle is rigorously controlled by multiple cellular factors [13,14,15]. It has been reported that Cyclin D-CDK4/6, Cyclin E-CDK2, and Cyclin A-CDK2 complexes regulate G1 progression and S phase entry [16]. The Cyclin B-CDK1 complexes, also known as MPF (mitosis promoting factor), play key roles in controlling G2 progression and M phase entry [17,18]. The contents of MPF are related to Cyclin B levels, whereas MPF activity is associated with the Wee1/mik1-mediated phosphorylation of Thr14/Tyr15 in CDK1 and cdc25-mediated dephosphorylation in the same amino acid sites [19,20,21,22]. During infection, viruses interfere with the cell cycle progression largely by deregulating cell cycle checkpoints at multiple levels of gene expression, including transcription, post-transcriptional processing, translation, post-translational modification, and protein stabilization [23]. Interactions between viral proteins and cyclins or CDKs (cyclin-dependent kinases, CDKs) affect the activity, expression levels, and redistribution of cyclins or CDKs [24,25,26,27]. Interestingly, some viruses (e.g., *Herpesvirus saimiri*) also encode cell cycle-related proteins to antagonize cell cycle checkpoints [28,29,30,31]. Therefore, cell cycle arrest is complicated, and the mechanism by which viruses manipulate the cell cycle progression still requires exploration.

Baculoviruses, which are highly pathogenic in insects, are circular, supercoiled, double-stranded DNA viruses. S or G2/M arrest occurs when insect cell lines are infected with baculoviruses (e.g., *Autographa californica* nuclear polyhedrosis virus (AcMNPV)) [32,33,34]. In addition, baculoviral genes, such as *IE-2* and *ODV-EC27*, are actively involved in the regulation of S and G2/M phase progression [35,36]. All findings suggest that baculoviruses actively manipulate the cell cycle via multiple viral genes. However, whether S or G2/M arrest is advantageous for viral replication remains poorly understood. Bombyx mori nuclear polyhedrosis virus (BmNPV), a typical member of the baculoviruses family, specifically infects the silkworm (Bombyx mori) and annually causes serious economic losses to the silkworm industry [37]. BmNPV encodes a 34 kDa protein (ORF120) which is a cyclin homolog with 48% homology to BmCyclin B and is related to BmNPV-mediated G2/M arrest in BmN (Bombyx mori-derived cell line) cells [34]. Based on these previous findings, we chose BmNPV to further explore the mechanism of baculovirus-mediated G2/M arrest in BmN-SWU1 cells. We found that G2/M phase arrest by BmNPV conferred the most advantageous cellular environment for BmNPV multiplication. We also demonstrated that BmNPV IAP1 interacted with BmCDK1, and that BmNPV *iap1* was involved in G2/M arrest. These findings improve our understanding of BmNPV pathogenesis and describe a new mechanism for baculovirus-mediated regulation of the cell cycle.

## 2. Materials and Methods

### 2.1. Cells and Virus Infection

The BmN-SWU1 cell line which derived from ovarian tissues of Bombyx mori [38], was cultured at 27 °C in TC-100 insect medium (United States Biological, Swampscott, MA, USA) supplemented with 10% fetal bovine serum (Biological Industries, Beit Haemek, Israel). Bombyx mori nucleopolyhedrovirus (BmNPV) (GenBank Accession No. NC001962.1) was inoculated onto a monolayer of cells at a multiplicity of infection (MOI) of 1.0. The inoculation at 1 h was defined as 0 h post infection (h p.i.). Subsequently, infected cells were cultured with fresh complete medium for the subsequent experiments.

### 2.2. Plasmid Construction and Transfection

The plasmids, pIZ-V5/His-*eGFP*, pIZ-*BmCDK1*-Flag, pIZ-BmNPV *iap1*-HA, pIZ-BmNPV *iap1*-*eGFP*, and psl1180-sgBmNPV *iap1*-Cas9-Flag, were constructed as described below. Briefly, the target sequences were amplified using PCR. These fragments were cloned into pIZ-V5/His or psl1180 vectors (Invitrogen, Shanghai, China, USA) by corresponding restriction enzyme digestion and T4 DNA ligation enzyme (Takara, 2011A). The positive clones were verified by sequencing. *BmCyclin B*-RNAi (RNA-interfering) and *BmCDK1*-RNAi vectors were prepared using the following method: endogenous miR RNAi sequences were predicted by online software BLOCK-ITTM RNAI Designer (http://rnaidesigner.invitrogen.com/rnaiexpress/sort.do, accessed on 10 November 2021). Three miR RNAi sequences were selected to replace silkworm endogenous microRNA backbones, bmo-mir-279 [39]. The three backbones were linked through a linker (linker sequence: GGTTCGTCGCCGAGAAATTG). The complete sequences were synthesized and cloned into pIZ-V5/His-*eGFP* vector (GenScript, Nanjing, China). The recombinant vBm (BmNPV)-*BmCDK1* was constructed as follows: BmCDK1 was cloned into pFastBac1 vector, and then recombinant vBm-*Bm*CDK1 was constructed by Bac-to-Bac, as described previously [40]. The promoter used to overexpress *BmCDK1* was from polyhedron promoter of pFastBac1 vector. PCR primers are shown in Table 1. Transfection of BmN-SWU1 cells was performed with Trans IT (R)-Insect Transfection Reagent (Mirus, MIR6106).

### 2.3. Cell Cycle Analysis

Cell cycle distribution was analyzed using Cell Cycle and Apoptosis Analysis Kit (Beyotime, Shanghai, China, C1025). BmN-SWU1 cells were collected, rinsed with sterile phosphate-buffered saline (PBS, pH 7.2) three times, and then fixed in 70% cold ethanol for 24 h at 4 °C. Then, the cells were stained with propidium (PI) and the fluorescence intensity was detected by a flow cytometer (Becton Dickinson, Franklin Lakes, NJ, USA).

### 2.4. MTS Assay

Detection of cell proliferation ability was performed using CellTiter 96^®^ AQueous One Solution Cell Proliferation Assay (MTS) (Promega, Madison, WI, G3582). In short, samples were collected at different time points post infection. The samples were cultured in 100 μL fresh medium supplemented with 100 μL CellTiter 96^®^ Aqueous single solution for 1–4 h in 96-well plates. Then, the absorbance was detected at 490 nm by Multi-Plate Reader (Biotek, Winosky, VT, USA).

### 2.5. Immunofluorescence

BmN-SWU1 cells were first seeded in a 24-well plate, and then transiently transfected or infected with BmNPV. At the indicated times, cells were fixed (4% paraformaldehyde, 15 min), permeabilized (1% Triton X-100 (Beyotime, Shanghai, China, ST677), 15 min), blocked (3% bovine serum albumin (BSA) and 10% sheep serum in PBS (blocking solution), 37 °C, 1 h), incubated with primary antibodies (1:200, 1.5 h, 37 °C), and incubated with secondary antibodies (1:500, 1 h, 37 °C) [41]. Triton X-100 (Beyotime, Shanghai, China, ST797), 4% paraformaldehyde (biosharp, Guangzhou, China, BL539A), BSA (Beyotime, Shanghai, China, ST023), sheep serum (ZSGB-BIO, ZLI-9021), primary antibodies (anti-Tubulin (Rabbit) (Beyotime, Shanghai, China, AF0001), anti-Brdu (mouse) (Roche, Mannheim, Germany, 11444611001), anti-Flag (mouse) (Abmart, Shanghai, China, M20008H), and anti-HA (Rabbit) (Invitrogen, Shanghai, China, 71–5500)), second antibodies (Alexa 555-conjugated donkey anti-rabbit antibody (Invitrogen, Shanghai, China, A32732), and Alexa 488-conjugated donkey anti-mouse antibody (Invitrogen, Shanghai, China, A32723)) were used in the present study.

### 2.6. BrdU or EdU Incorporation

Cells were labeled with BrdU at 5 mg/mL for 2 h, then analyzed with an immunofluorescence assay in accordance with the instructions of the 5-Bromo-2′-deoxy-uridine Labeling and Detection Kit III (Roche, Mannheim, Germany, 11444611001). Cells were labeled with EdU at 1 mM for 2 h, and were subjected to click reaction in accordance with the instructions of the BeyoClick^TM^ EdU Cell Proliferation Kit (Beyotime, Shanghai, China, C0075S).

### 2.7. Cell Synchronization

Aphidicolin (Sigma, Saint Louis, MO, USA, A0781) (5 mg/mL, 24 h) was used to synchronize BmN-SWU1 cells in the G1 phase. Hydroxyurea (Sigma, Saint Louis, MO, USA, H8627) was used to synchronize BmN-SWU1 cells in the S phase. Cells were incubated with 1 mM hydroxyurea for 16 h and cultured in hydroxyurea-washed medium for 7 h. Nocodazole (Sigma, Saint Louis, MO, USA, M1404) (10 mg/mL, 24 h) was used to synchronize BmN-SWU1 cells in the G2/M phase.

### 2.8. TCID50

BmN-SWU1 cells were infected with vBm-*eGFP* expressing EGFP and the supernatant containing progeny virus was collected. The supernatant was serially diluted 10-fold, and 100 μL of each dilution was added into 96-well plates. The viral titer of progeny virus was determined by recording green fluorescence at different time points post infection.

### 2.9. Quantitative Real Time-PCR (qRT-PCR)

Cellular RNA was extracted using Total RNA Kit II (OMEGA, Beijing, China, R6834-02). Then, PrimeScript^TM^ RT Reagent Kit (TAKARA, Dalian, China, RR047B (Ax4)) was used to reverse transcribed RNA into cDNA. Wizard^®^ Genomic DNA Purification Kit (Promega, Madison, WI, USA, A1120) was used to extract cellular genomic DNA. The primer sequences used in target gene amplification are listed in Table 1. Eukaryotic translation initiation factor 4A (silkworm microarray probe ID: sw22934) was used as an internal control, and *gp41* was used to quantify viral genome copies. qRT-PCR was conducted using Hieff^TM^ qPCR SYBR Green Master Mix (Yeasen, Shanghai, China, 11201ES08) and analyzed with CFX Connect Real-Time PCR Detection System (Bio-Rad, Hercules, CA, USA). PCR reaction procedure was as follows: 95 °C for 5 min, followed by 44 cycles of 95 °C for 10 s and 60 °C for 30 s.

### 2.10. SDS-PAGE and Western Blotting

Cells were lysed using cell lysis buffer for Western blot analysis and IP (Beyotime, Shanghai, China, P0013) supplemented with 1% PMSF (Beyotime, Shanghai, China, ST505). BCA Protein Assay Kit (Beyotime, Shanghai, China, P0010) was used to measure protein concentration. 12% SDS-PAGE was used to resolve proteins. Proteins were electrophoretically transferred onto PVDF Western blotting membranes (Roche, Mannheim, Germany, 3010040001). Proteins in the membranes were analyzed by enhanced chemiluminescence (ECL) reagents (Yeasen, Shanghai, China, 36208ES76) as described previously [42].

Primary antibodies were anti-Flag (mouse) (Abmart, Shanghai, China, M20008H), anti-Tubulin (Rabbit) (Beyotime, Shanghai, China, AF0001), and anti-BmCyclin B (Rabbit), which was previously prepared in our laboratory. HRP-labeled goat anti-mouse (or anti-rabbit) IgG (H + L) was applied as secondary antibody. Protein markers were Blue Plus^TM^ protein marker (TransGen, Beijing, China, DM111-01) and EasySee^TM^ Western Marker (TransGene Biotech, Beijing, China, DM201-02).

### 2.11. Co-Immunoprecipitation and LC-MS/MS Analysis

BmN-SWU1 cells were lysed using 1 mL of cell lysis buffer for Western blot analysis and IP. Protein A+G magnetic (50 μL) (Yeasen, Shanghai, China, 36417ES08) and antibody (α-Flag monoclonal antibody or mouse IgG) (5 μL), were mixed and incubated for 1 h at room temperature. Protein samples were incubated with above mixtures for 2–3 h at room temperature. After incubating, the beads were collected, washed with PBS containing 5% Tween (PBST) (Beyotime, Shanghai, China, ST825), and incubated with 5× loading buffer (Beyotime, Shanghai, China, P0015L) at 100 °C for 10 min. After co-immunoprecipitation, protein samples were resolved using 12% SDS-PAGE and the gels were stained using silver staining. Finally, LC–MS/MS analysis was performed as described previously [43].

### 2.12. Statistical Analyses

Statistical differences were analyzed by student’s *t*-test using GraphPad Prism 5.0 software. When the *P* value was ≥0.05, the difference was considered as not significant (ns). When the *P* value was <0.05, the difference was considered as significant. When the *P* value was <0.01, the difference was considered as highly significant.

## 3. Results

### 3.1. Regulation of the Cell Cycle in BmNPV-Infected Cells

**Figure 1:**
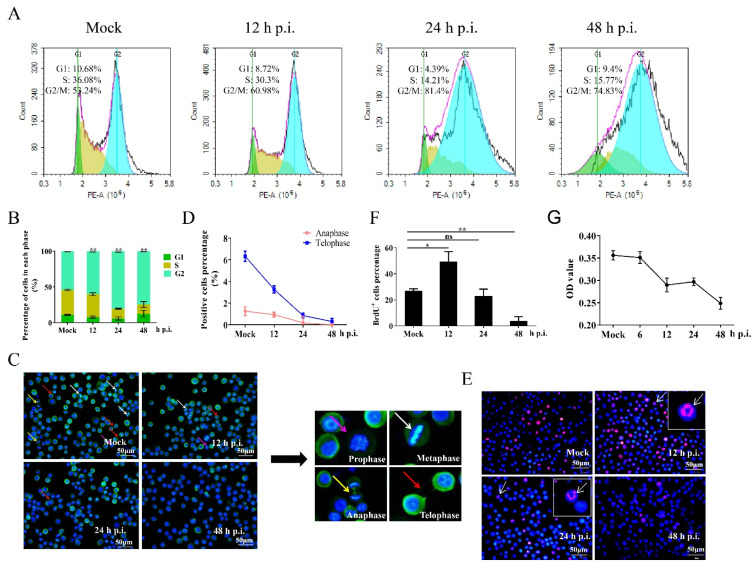
Regulation of the cell cycle in BmNPV-infected cells. BmN-SWU1 cells were infected with 1 MOI of BmNPV, and the cell cycle was analyzed at mock infection and different time points post infection. (**A**,**B**) Cellular DNA was stained by propidium iodide (PI), and the cell cycle distribution was determined in mock and infected cells by flow cytometry. The data were analyzed as mean ± SD from three independent experiments. (**C**) Mitotic stage was analyzed by Hoechst33342 (blue fluorescence) and tubulin (green fluorescence) staining. Pink arrow indicates prophase, white arrow indicates metaphase, yellow arrow indicates anaphase, and red arrow indicates telophase. Scale bar: 50 μm. (**D**) Statistical analysis of the proportion of anaphase and telophase cells. (**E**,**F**) Comparison and statistical analysis of BrdU-positive cells. Anti-5-bromodeoxyurdine (anti-BrdU)-labeled BmN-SWU1 cells infected with or without BmNPV. White arrow indicates cells containing replicating viral genomes. Scale bar: 50 μm. (**G**) Cell proliferation profile of BmN-SWU1 cells analyzed by MTS. OD: optical density. Statistical differences were analyzed with Student’s *t*-test (* *p* < 0.05 and ** *p* < 0.01).

Many viruses induce a delay, blockade, or prolongation in the cell cycle to promote viral yield [44,45,46]. BmN-SWU1 cells are susceptible to BmNPV. Therefore, the change in cell cycle was first assessed in BmNPV-infected BmN-SWU1 cells. We used flow cytometry (FCM) to determine cell cycle distribution at different time points post infection. The results indicate that there was no obvious effect before 9 h p.i. (Figure S1A,B). However, G2/M phase cells were significantly accumulated at 12 h p.i. (Figure 1A,B). In addition, cells in prophase, metaphase, anaphase and telophase were observed in non-infected cells labeled by Hoechst33342 and anti-tubulin, while only a few of cells in anaphase and telophase were observed 24 h and 48 h p.i. (Figure 1C). Statistical analyses showed that the proportion of cells in anaphase and telophase was markedly reduced 12 h p.i. compared to the uninfected counterpart, suggesting that BmNPV infection inhibited the progression to M phase (Figure 1D).

Baculovirus-infected cells display a structural characteristic known as virogenic stroma, which is located near the center of the nucleus and has been identified as the site of viral genome replication using 5-bromodeoxyuridine (BrdU) labeling [47,48]. To determine the effects of BmNPV infection on cellular DNA replication, mock and infected BmN-SWU1 cells were labeled with BrdU. The BrdU signal was markedly increased at 12 h p.i., but was not significantly changed at 24 h p.i. and was significantly decreased at 48 h p.i. (Figure 1E,F). The majority of BrdU signaling was elicited by replication of BmNPV DNA 12 h and 24 h p.i., indicating that BmNPV infection greatly repressed cellular DNA synthesis. The proliferation of BmNPV-infected cells was analyzed using MTS. Our results reveal that cell proliferation was significantly inhibited 12 h p.i. (Figure 1G), which agreed with the results obtained using the TC20^TM^ Automated Cell Counter (Figure S1C). These results demonstrate that BmNPV induces G2/M arrest, and inhibits cellular DNA replication and cell proliferation.

### 3.2. Cells in G2/M Phase Create a Favorable Environment for BmNPV Multiplication

**Figure 2:**
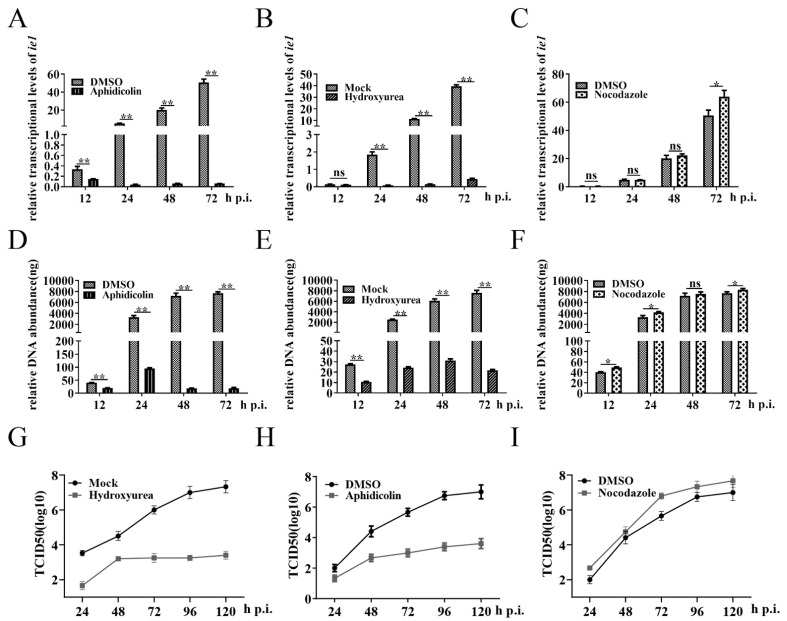
Cells in the G2/M phase create a favorable environment for BmNPV multiplication. The cell cycle of BmN-SWU1 cells was synchronized in the G1 phase by aphidicolin (5 mg/mL, 24 h), in the S phase by hydroxyurea (cells were cultured with 1 mM hydroxyurea for 16 h and without hydroxyurea for 7 h), and in the G2/M phase by nocodazole (10 mg/mL, 24 h). Then, cells were infected with 1 MOI of BmNPV. At 1 h post infection, cells were cultured in fresh culture medium supplemented with aphidicolin, hydroxyurea, and nocodazole at predetermined concentrations. (**A**–**C**) The mRNA levels of *ie1* in aphidicolin-, hydroxyurea-, or nocodazole-treated cells were analyzed using qRT-PCR. (**D**–**F**) Viral genome copies were detected in aphidicolin-, hydroxyurea-, or nocodazole-treated cells. (**G**–**I**) Viral titer analysis of progeny viruses in aphidicolin-, hydroxyurea-, or nocodazole-treated cells by TCID50. The data are presented as the mean ± SD of three independent experiments. Statistical differences were analyzed with Student’s *t*-test (^ns^ *p* ≥ 0.05, * *p* < 0.05 and ** *p* < 0.01).

To explore whether G2/M arrest was critical for the proliferation and replication of BmNPV, we used aphidicolin, hydroxyurea, and nocodazole to synchronize BmN-SWU1 cells in the G1, S, and G2/M phase, respectively (Figure S2A–C). The results obtained using qRT-PCR suggest that G2/M phase cells supported the expression of *ie1* (a BmNPV immediate gene that was necessary for the proliferation and replication of BmNPV [49]) and viral DNA replication compared to the control cells (Figure 2C,F). However, the expression of viral *ie1* gene and viral DNA replication were significantly inhibited when BmNPV infected G1 and S phase cells (Figure 2A–E). Through TCID50 analysis, we found that the viral titer increased in the G2/M phase cells, but decreased in the G1 and S phase cells (Figure 2G–I). These results demonstrate that the G2/M phase, but not the G1 and S phases, is advantageous for the proliferation and replication of BmNPV.

### 3.3. G2/M Arrest by BmCDK1 and BmCyclin B Enhances BmNPV Proliferation

**Figure 3:**
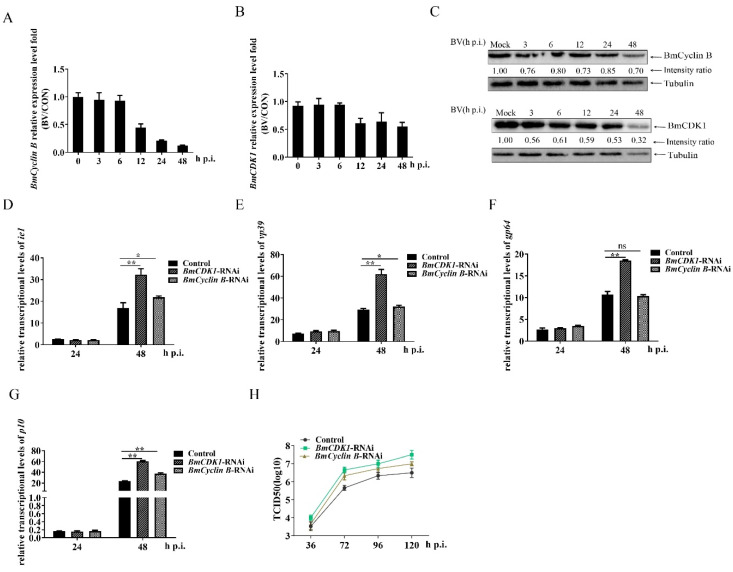
G2/M arrest by *BmCDK1* and *BmCyclin B* enhances BmNPV proliferation. BmN-SWU1 cells were infected with 1 MOI of BmNPV. (**A**,**B**) The relative expression levels of *BmCyclin B* and *BmCDK1* in different periods of BmNPV infection (BV/CON represented as the ratios of the mRNA levels of *BmCyclin B* or *BmCDK1* in infected cells to that in uninfected cells). (**C**) Western blotting was used to analyze the amount of BmCyclin B and BmCDK1. The protein band density ratios of BmCyclin B and BmCDK1 were calculated by protein band density analysis using Image Lab. Tubulin was adopted as the loading control. (**D**,**E**) Expression levels of *ie1* and *vp39* after RNAi silencing of *BmCyclin B* and *BmCDK1*. Cells transfected with pIZ-*eGFP* were adopted as the control. (**F**,**G**) Expression levels of *gp64* and *p10* after RNAi silencing of *BmCyclin B* or *BmCDK1*. (**H**) Viral titer analysis of progeny viruses after RNAi silencing of *BmCyclin B* or *BmCDK1* by TCID50. The data are presented as the mean ± SD of three independent experiments. Statistical differences were analyzed with Student’s *t*-test (^ns^ *p* ≥ 0.05, * *p* < 0.05, and ** *p* < 0.01).

*Cyclin B* and *CDK1* are the key genes controlling G2 progression and M phase entry [19]. To explore the molecular mechanisms of BmNPV-induced G2/M arrest, we first analyzed the expression of *BmCyclin B* and *BmCDK1* at predetermined time points post infection. The results of qRT-PCR analysis indicated that the expression levels of *BmCyclin B* and *BmCDK1* tended to be reduced 12 h p.i. (Figure 3A,B). Immunoblotting revealed that the amounts of BmCyclin B and BmCDK1 were already reduced at 3 h p.i. compared to the uninfected cells (Figure 3C). To further investigate whether BmNPV augmented its multiplication by inducing G2/M phase arrest via *BmCyclin B* and *BmCDK1*, we constructed microRNA-mediated *BmCyclin B* and *BmCDK1* RNA-interfering (RNAi) recombinant plasmids (interference efficiency is shown in Figure S2D). FCM analysis showed that RNAi-mediated reduction of *BmCyclin B* and *BmCDK1* expression induced the significant accumulation of G2/M phase cells compared with the control (Figure S2E,F). The results obtained using qRT-PCR indicated that the RNAi-mediated reduction of *BmCyclin B* expression increased the relative expression levels of *ie1*, *vp39* (a BmNPV late gene that is essential for the production of infection viruses [49]), and *p10* (a BmNPV very late gene [50]), but there was no difference in the expression of *gp64* (a BmNPV early gene that plays essential roles in initiating the entry of budded virus [51]) between the RNAi group and control group. The RNAi-mediated reduction of *BmCDK1* expression markedly increased the expression of *ie1*, *vp39*, *gp64*, and *p10* (Figure 3D–G). TCID50 analysis showed that the RNAi-mediated reduction of *BmCDK1* and *BmCyclin B* increased viral titer compared to the control group. These findings suggest that BmNPV induces G2/M phase arrest via *BmCyclin B* and *BmCDK1*, thereby promoting BmNPV proliferation.

### 3.4. BmNPV IAP1 Interacts with BmCDK1

**Figure 4:**
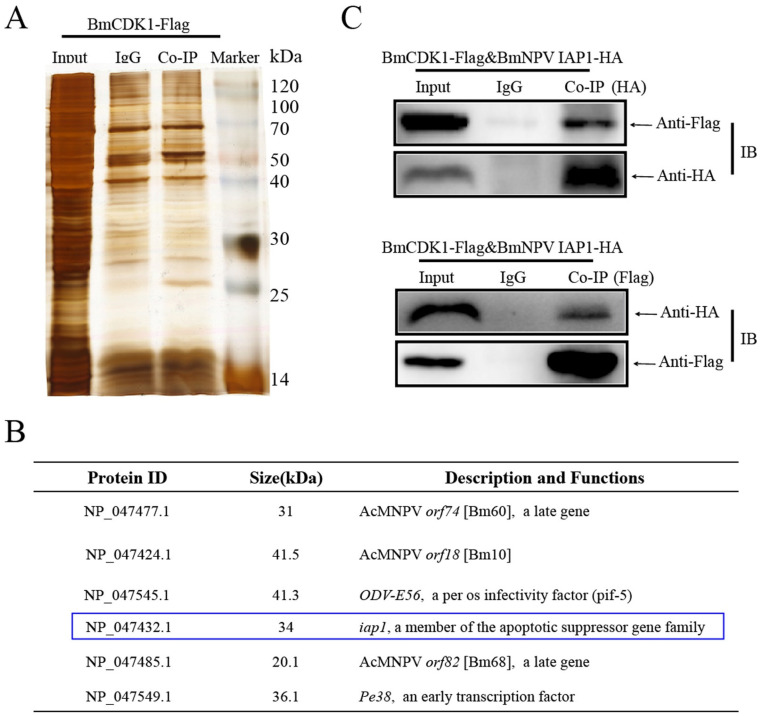
BmNPV IAP1 interacts with BmCDK1. (**A**) BmCDK1 was overexpressed by recombinant BmNPV (vBm-*BmCDK1*). BmN-SWU1 cells were infected with 1 MOI of vBm-*BmCDK1*. Co-immunoprecipitation (Co-IP) was performed 48 h p.i. Marker: protein molecular weight marker; Input: input cell lysates; IgG: IP with mouse IgG; Co-IP: IP with α-Flag monoclonal antibody. IgG and Co-IP bands of three independent experiments were combined and sent for LC–MS/MS analysis. IgG groups were applied as the negative control. (**B**) Candidate viral proteins interacting with BmCDK1 by LC–MS/MS. Blue box indicates the target protein for the subsequent investigations. (**C**) Co-IP of BmCDK1 and BmNPV IAP1. pIZ-BmNPV *iap1*-HA was transfected into BmN-SWU1 cells, and cells were infected with 1 MOI of vBm-*BmCDK1* 48 h post transfection. Co-IP was analyzed with Western blotting 48 h p.i.

Virus–host interaction is a universal strategy used by viruses to arrest the host cell cycle progression [24]. Based on the knowledge that *BmCDK1* influences G2/M progression and BmNPV yield, we overexpressed *BmCDK1* by recombining it with BmNPV genome (vBm-*BmCDK1*). Then, we adopted Co-IP (co-immunoprecipitation) to find out viral proteins interacting with BmCDK1 (Figure 4A). Our screen revealed six viral protein candidates (Figure 4B). Among these proteins, BmNPV *iap1* was selected as the target gene due to the fact that cellular *iaps* was shown to affect host mitosis [52,53,54]. Thus, we used Co-IP experiments to confirm the interaction between BmCDK1 and BmNPV IAP1. We detected BmNPV IAP1^HA^ in the group labeled with an α-Flag monoclonal antibody, while BmCDK1^Flag^ was also detected in another group labeled with an α-HA monoclonal antibody. No target bands were detected in the IgG control groups (Figure 4C). These results indicate that BmCDK1 interacts with BmNPV IAP1 during BmNPV infection.

### 3.5. BmNPV iap1 Regulates the Cell Cycle via BmCDK1

**Figure 5:**
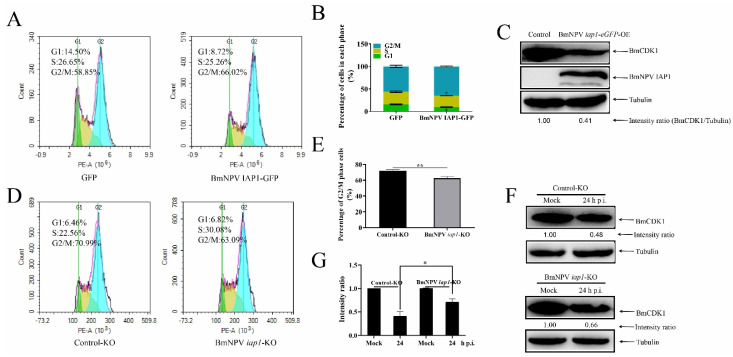
BmNPV *iap1* regulates the cell cycle via *BmCDK1*. (**A**,**B**) Comparison of cell cycle distribution in pIZ-*eGFP* and pIZ-BmNPV *iap1*-*eGFP* groups. The pIZ-*eGFP* group was used as a negative control. The data were analyzed as mean ± SD from three independent experiments. (**C**) The amount of BmCDK1 was analyzed after co-transfecting with pIZ-*BmCDK1*-Flag, pIZ-BmNPV *iap1*-HA, pIZ-*BmCDK1*-Flag, and pIZ-V5/His, successively. Tubulin was adopted as the loading control. (**D**,**E**) 48 h post transfection with control-KO and BmNPV *iap1*-KO, cells were infected with BmNPV. The percentage of G2/M phase cells 24 h p.i. were presented as mean ± SD from three independent experiments. (**F**) The amount of BmCDK1 protein were detected in no-infected and 24 h p.i. cells using Western blotting. Tubulin was adopted as the loading control. The protein band density ratios of BmCDK1 were calculated by protein band density analysis using Image Lab. (**G**) Results shown with graphs representing the intensity of the BmCDK1/tubulin ratio. Statistical differences were analyzed with Student’s *t*-test (* *p* < 0.05 and ** *p* < 0.01).

To investigate whether BmNPV *iap1* could regulate host cell cycle progression into the G2/M phase, we constructed a BmNPV *iap1* overexpression plasmid that was fusion-expressed with GFP as well as a BmNPV *iap1* knockdown plasmid (BmNPV *iap1* was knocked down by the CRISPR/Cas9 gene editing system as shown in Figure S3A). The results obtained via FCM showed that the overexpression of BmNPV *iap1* gene displayed significant accumulation of G2/M phase cells from 56.53 ± 2.54% to 64.88 ± 1.00%. On the other hand, overexpressed BmNPV *iap1* gene reduced the proportion of G1 phase cells from 15.59 ± 1.00% to 9.49 ± 0.68%, but did not affect S phase progression (Figure 5A,B). There was a lower proportion of G2/M phase cells in the BmNPV *iap1*-KO group compared with in the control-KO group (Figure 5D,E). We also performed qRT-PCR and immunoblotting analyses to examine whether BmNPV *iap1* regulated the expression of *BmCDK1*. As shown in Figure S3B,C, the BmNPV *iap1* gene was not involved in regulating the transcription of *BmCDK1*. However, the overexpression of BmNPV *iap1* gene significantly decreased the amount of BmCDK1, and the knockdown of BmNPV *iap1* gene inhibited the BmNPV-mediated decrease of BmCDK1 amount correspondingly (Figure 5C,F,G). Taken together, these results confirm that BmNPV *iap1* is involved in G2/M phase arrest by *BmCDK1* during BmNPV infection.

## 4. Discussion

Many viruses utilize the host cell’s macromolecular synthesis mechanism, energy, and specific intracellular environment to regulate the cell cycle progression [55,56,57,58,59,60,61]. To date, the relationship between Baculoviruses and the cell cycle is only partially understood; few studies have examined whether a specific cell cycle phase may be advantageous for viral replication. Our current study revealed that BmNPV induced G2/M arrest to benefit its proliferation and replication 12 h p.i. (Figure 2). Arrest in the G2/M phase confers several advantages for BmNPV replication. Cellular DNA replication was inhibited to enable BmNPV DNA replication 12 h p.i. (Figure 1E). The viral genes involved in BmNPV DNA synthesis have been identified as *dnapol*, *helicase*, *lef1*, *lef2*, *lef3*, *dbp*, *lef11*, and *lef7* [62]. Thus, it is possible that BmNPV-mediated G2/M arrest prevents the host from utilizing viral components. Our results showed that BmNPV inhibited the progression to the M phase 12 h p.i. to prevent the sequestration of materials needed for viral replication (Figure 1C,D). A previous study suggest that pronounced inhibition of interferon (IFN) and IFN-stimulated genes expression appeared in the G2/M phase cells, indicating mitotic transcriptional inhibition of the G2/M phase to circumvent an antiviral response [46]. Moreover, G2/M arrest may also be advantageous for viral transport due to the availability of microtubules or the mitotic spindle. Further investigations are needed to determine the underlying reasons for BmNPV-mediated G2/M arrest, which may help explain pathogenic characteristics of BmNPV and identify new antiviral targets.

**Figure 6:**
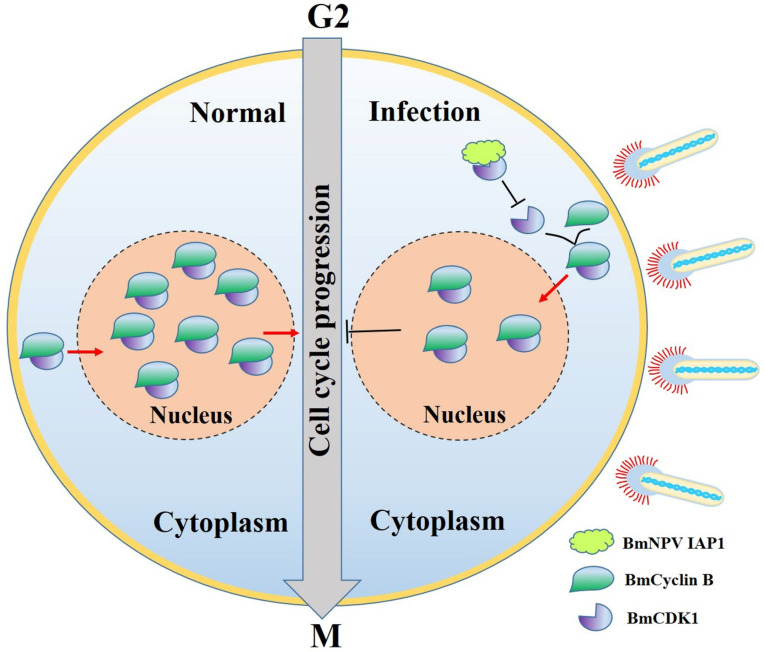
Schematic diagram of the molecular mechanism of BmNPV-mediated cell cycle arrest. Following BmNPV infection, BmNPV IAP1 interacts with BmCDK1 and inhibits the expressions of BmCDK1. These events result in reduced levels of BmCyclin B-BmCDK1 complexes and subsequent G2/M arrest.

Viruses utilize various strategies to manipulate the cell cycle, highlighting the necessity of further exploring the mechanism involved in BmNPV-mediated G2/M arrest. BmNPV exerted no significant effects on the cell cycle before 9 h p.i. (Figure S1A,B), implying that G2/M arrest was independent of virus entry and may be related to viral replication or viral-protein manufacturing [50]. BmNPV caused an accumulation of G2/M phase cells by suppressing the expression of *BmCyclin B* and *BmCDK1* (Figure 1 and Figure 3). The mRNA levels of *BmCyclin B* and *BmCDK1* tended to be reduced 12 h p.i., which was a consistent response time for a global shutoff of host transcription 12–18 h post infection in baculoviruses [63,64]. However, the mechanism of baculovirus-mediated global shutoff of host transcription remains unclear. Transactivational regulators of baculovirus are extremely important for the effective transcription of viral genes [62]. Among them, the immediate early gene, *ie1*, may participate in the negative regulation of some genes [65]. Thus, the mRNA levels of *BmCyclin B* and *BmCDK1* may be negatively regulated by *ie1*. Previous studies have shown that G2/M arrest is accompanied by upregulated expression of *Cyclin B* and *CDK1* [33,66]. This discrepancy may stem from BmNPV-mediated inhibition of the G2 to M phase transition by reducing the accumulation of active BmCyclin B-BmCDK1 complexes, while other viruses may behave in an opposite way. Therefore, we hypothesized that certain BmNPV proteins can induce G2/M arrest by interacting with BmCyclin B or BmCDK1. The results in the present study confirmed this hypothesis (Figure 4 and Figure 5). As shown in Figure 6, BmNPV IAP1 specifically interacts with BmCDK1 and inhibits the expression of BmCDK1 after BmNPV infection, thereby reducing the levels of BmCyclin B-BmCDK1 complexes within the nucleus and leading to G2/M arrest. BmNPV ORF120 is a cyclin homolog with 48% homology to BmCyclin B and has an associated kinase activity with histone H1 [34], implying the role of BmNPV *ORF120*-mediated G2/M arrest. The transcription start time of BmNPV *ORF120* is 6 h p.i., and that of BmNPV *iap1* is 12 h p.i. [50], which can interpret why the extent of G2/M phase arrest induced by BmNPV infection was much greater than that by induced by overexpressing BmNPV *iap1* (Figure 1A,B and Figure 5A,B). In addition, unidentified candidates also suggest that BmNPV *iap1* is just one of the BmNPV genes that induce G2/M arrest.

It is well-known that cellular inhibitor-of-apoptosis (IAP) proteins play important roles in blocking apoptosis. IAP proteins have been exploited by multiple viruses to enable the increased efficiency of viral replication [67,68,69,70]. Moreover, increasing evidence has shown that cellular *iaps* regulates the process of cell cycle. For example, IAPs disrupted chromosomal passenger complex (CPC) localization to the centromeres and interacted with the serine/threonine-protein kinase polo, indicating that *iaps* were involved in regulation of the cell cycle [52,53]. In medulloblastoma cells, *ciap1/2* or *xiap* regulated G2/M phase progression via Cyclin B1-CDK1 and Cyclin A-CDK1/2 [71]. The treatment of human laryngeal carcinoma cells with *Xiap*-shRNA induced G0/G1 phase arrest [72]. These findings implies that viral *iaps* also play crucial roles in regulating the cell cycle, and are involved in crosstalk between apoptotic and cell cycle. Here, we confirmed that the overexpression of BmNPV *iap1* blocked the cell cycle in the G2/M phase via downregulation of BmCDK1 (Figure 5A–C). The CRISPR/Cas9 system has shown the ability to cleave viral genes in insect cells, and the system has been well-established in our lab [73]. However, it is complex to construct BmNPV knockout bacmids by the lambda red recombination system [49]. Therefore, the BmNPV *iap1* gene was knocked down by the CRISPR/Cas9 system in this study, and the results showed that BmNPV *iap1* regulated G2/M arrest via the amount of BmCDK1 corresponding to BmNPV infection (Figure 5D–G). Most virus-encoded IAPs possess C-terminal RING domain, baculovirus IAP repeat (BIR) domains, and a short N-terminal leader [74]. The C-terminal RING domain of cellular IAPs possesses E3-ubiquitin ligase activity [75]. Thus, the BmNPV *iap1*-mediated reduction of BmCDK1 probably occurs via the C-terminal RING domain of BmNPV *iap1*, which will be investigated in our future studies.

In conclusion, our data indicate that G2/M arrest increased the yield of BmNPV. The current study also suggests a novel function of BmNPV *iap1* for inducing cell cycle arrest. We will systematically investigate whether baculovirus *iaps* commonly regulates the cell cycle, identify the interaction of other viral proteins and BmCDK1, and explore the mechanism for BmNPV-related G2/M arrest in the follow-up investigations.

## Figures and Tables

**Table 1:** Primers used in current study.

Primers for Clone	Sequence
>*BmCDK1^Flag^*-F	F:ATTTGCGGCCGCATGGATTACAAGGATGACGACGATAAGGATGATTTCTTAAAGATAGAAAAGATCG
>*BmCDK1^Flag^*-R	R:TGCTCTAGATTACTTATCGTCGTCATCCTTGTAATCTACACTTTGAACAGAATCCGTGTC
>BmNPV *iap^HA^*-F	CGCGGATCCATGTACCCATACGATGTTCCAGATTACGCTAACGAGGACACTCCTCCGT
>BmNPV *iap^HA^*-R	TGCTCTAGACACCACAAATATTTTTATAAAATCG
>SgBmNPV *iap1*-F	AAGTGTGAAGCAGAAATAAAAAAT
>SgBmNPV *iap1*-R	AAACATTTTTTATTTCTGCTTCAC
Primers for qRT-PCR	Sequence
>*BmCyclin B*	F: TGTCAAAAATGTTATTCAGCC; R: TTTCCGTAAAGAGTCAGTTCC.
>*BmCDK1*	F: AGGGCTCCTGAGGTCTTACT; R: TGTTGGCGTTCTTAGCATTCT.
>*ie-1*	F: AAGAAGGAGGACGGCAGCAT; R: ATCTCGCCAGAAATCCAATAAAAC.
>*vp39*	F: CTAATGCCCGTGGGTATGG R: TTGATGAGGTGGCTGTTGC
>*gp64*	F: CACCATCGTGGAGACGGACTA R: CCTCGCACTGCTGCCTGA
>*p10*	F: TAGACGCCATTGCGGAAA F: CGGGCAAACCGTCCAAA
>*gp41*	F: ATGTTGATGTGCGGAAAGC; R: GTGGCGGAATCGGTGA.
>*sw22934*	F: TTCGTACTGGCTCTTCTCGT; R: CAAAGTTGATAGCAATTCCCT.
Primers for RNAi	Sequence
*BmCyclin B*-miRNA1	AATGTGCACATTCAACGCGGAA TTCGCGTTGAACTTGCACATT
*BmCyclin B*-miRNA2	GTTGCCGGCACAACTGCTGGAA TTCAGCAGTTGTAACGGCAAC
*BmCyclin B*-miRNA3	GCGTAGTGTGAAGCAACTAGTA TATAGTTGCTTCGTACTACGC
*BmCDK1*-miRNA1	TAAACGACACCGTAAGTACCT AGGTACTCGCGGTGTCGTTGTA
*BmCDK1*-miRNA1	CCCTTGGCGCTGGAAAGTTGCA TGAACTTTCCAGAACCAAGGG
*BmCDK1*-miRNA1	TCAACAACTCCATCTTGTAGCT AGTACAAGATGGCATTGTTGA

(The restriction-enzyme sites are underlined).

## Data Availability

The data presented in this study are available in insert article or supplementary material here.

## Supplementary Materials

The following are available online at https://www.mdpi.com/article/10.3390/insects12121098/s1, Figure S1: Cell-cycle analysis in BmN-SWU1 cells infected BmNPV. BmN-SWU1 cells were infected with 1 MOI of BmNPV, then cell cycle was analyzed at mock infection and different time points post infection. (A,B) Cellular DNA was stained by propidium iodide (PI), then cell-cycle distribution was determined in mock and infected cells by flow cytometry. The data were analyzed as mean ± SD from three independent experiments.. (C) Cell numbers were evaluated using TC20TM Automated Cell Counter (Bio-Rad, California, USA). (** *p* < 0.01). Figure S2: Analysis of cell-cycle perturbations. (A–C) Cell-cycle perturbations were analyzed after treating the cells with aphidicollin (5 mg/mL, 24 h), hydroxyurea (cells were cultured with 1mM hydroxyurea for 16 h and without hydroxyurea for 7 h) and nocodazole (10 mg/mL, 24 h), respectively. (D) Relative transcription levels of *BmCDK1* and *BmCyclin B* after treatment with RNAi. (E) Comparison of cell-cycle distribution in pIZ-*eGFP*, pIZ-*BmCDK1*-RNAi-*eGFP*, and pIZ-*BmCyclin B*-RNAi-*eGFP* groups. pIZ-*eGFP* group was used as a negative control. (F) The data were presented as mean ± SD from three independent experiments. Statistical differences were analyzed with Student’s t-test (** *p* < 0.01). Figure S3: BmNPV *iap1* has no effect on the mRNA levels of *BmCDK1*. (A) BmNPV *iap1* was edited with the CRISPR/Cas9 gene editing system. Briefly, BmNPV *iap1* was amplified by PCR, then the PCR product was cloned into the pMD19-T vector. The recombinant plasmids were sequenced using M13 primers. Knock out efficiency of BmNPV *iap1* was 41.67%. (B,C) Analyze the mRNA levels of *BmCDK1* after overexpression of BmNPV *iap1* and BmNPV *iap1* knock out via the CRISPR/Cas9 gene editing system. The data were presented as mean ± SD from three independent experiments. Statistical differences were analyzed with Student’s t-test ^(ns^ *p* ≥ 0.05). Figure S4: The whole blots of Figure 3C, Figure 4C, and Figure 5C,F.
