# Supplementary material for: Bombyx mori Nucleopolyhedrovirus (BmNPV) Induces G2/M Arrest to Promote Viral Multiplication by Depleting BmCDK1
Source: Insects. 2021 Dec 8;12(12):1098. doi: 10.3390/insects12121098 (PMC8708760; doi:10.3390/insects12121098)
Supplement: Supplementary file 1 [file insects-12-01098-s001.zip › insects-1418021-supplementary.pdf]

**Supporting information**

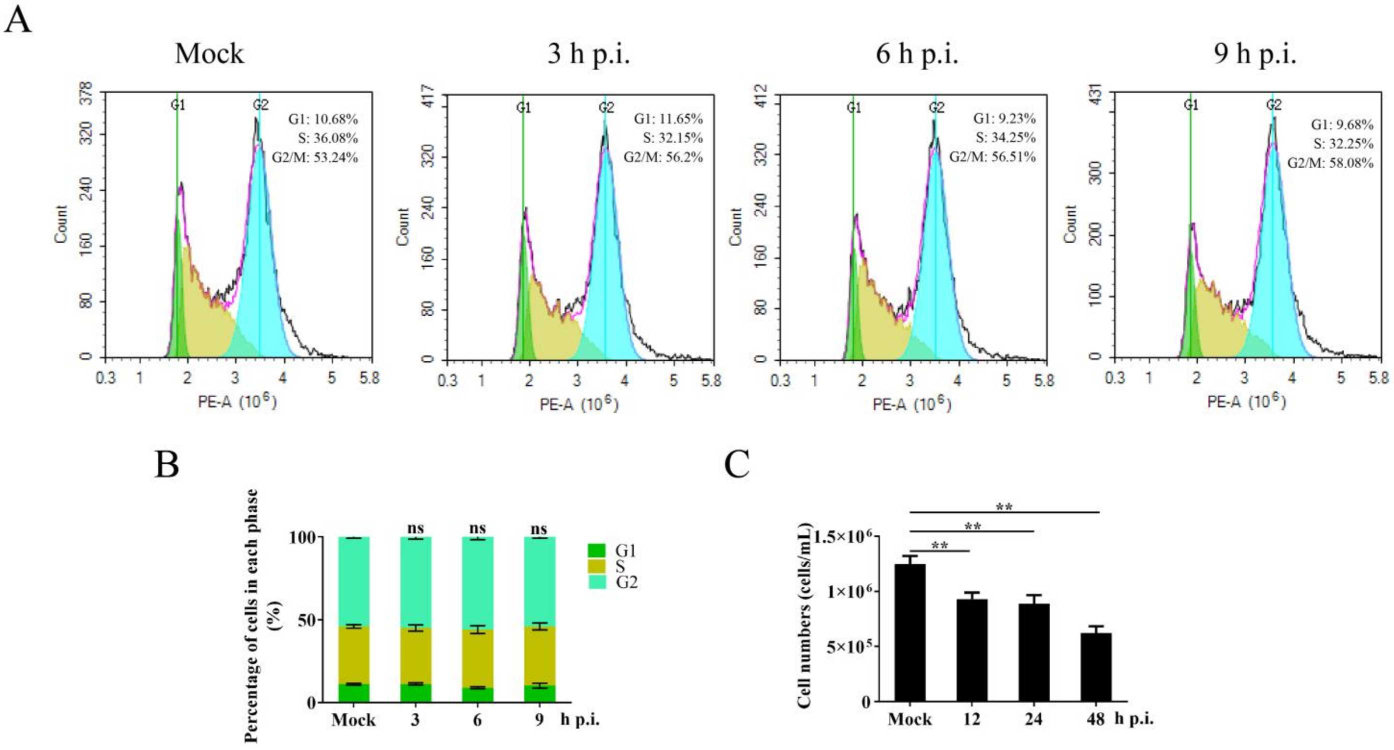


**Figure.S1 Cell-cycle analysis in BmN-SWU1 cells infected BmNPV.** (A-B) Comparison of cell-cycle distribution in mock and infected cells. (C) Cell numbers were evaluated using TC20TM Automated Cell Counter. (** *p*<0.01)

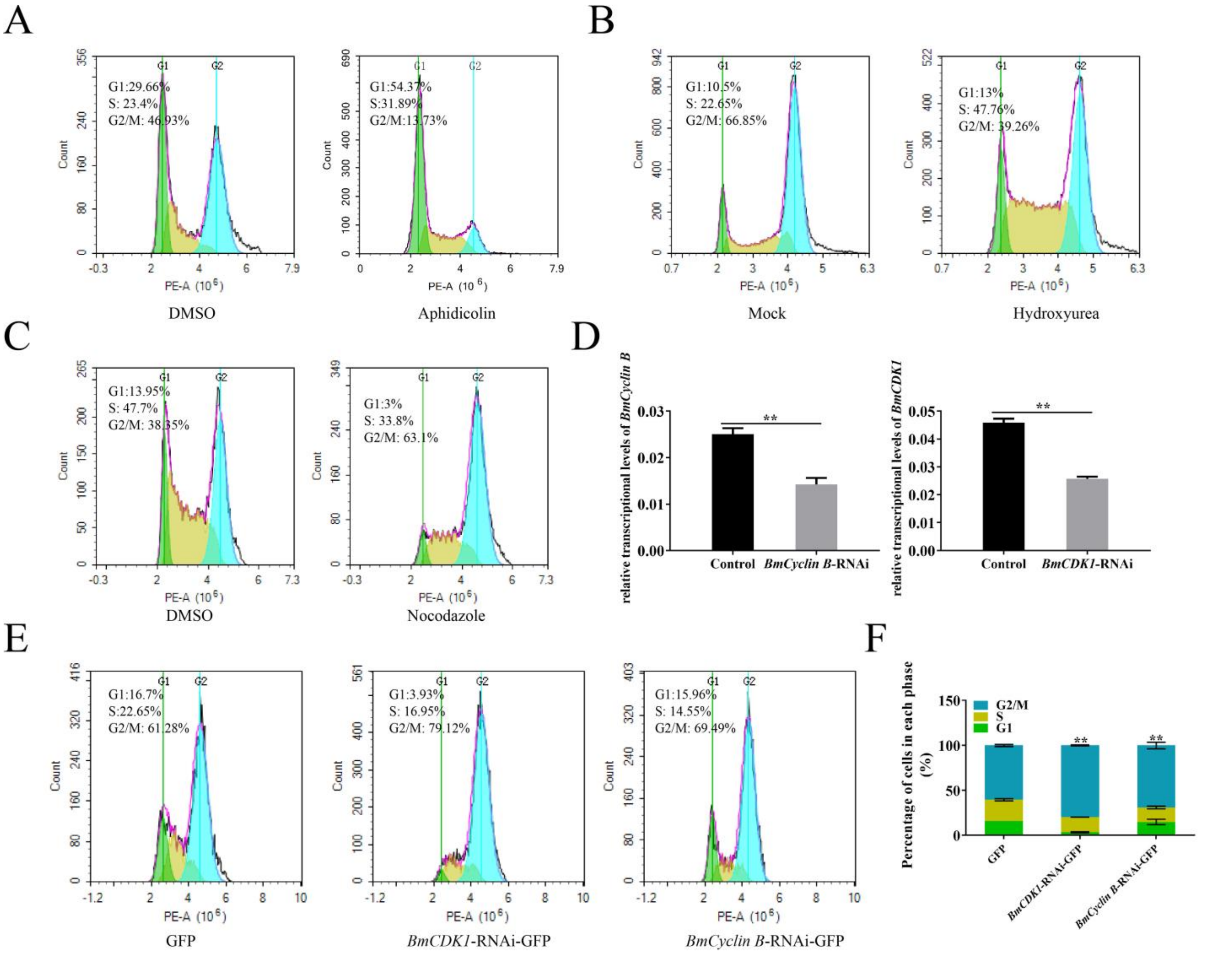


**Figure.S2 Analysis of cell-cycle perturbations.** (A–C) Cell-cycle perturbations were analyzed after treating the cells with aphidicollin, hydroxyurea and nocodazole, respectively. (D) Relative transcription levels of *BmCDK1* and *BmCyclin B* after treatment with RNAi. (E) Comparison of cell-cycle distribution in pIZ-*eGFP*, pIZ-*BmCDK1*-RNAi-*eGFP*, and pIZ-*BmCyclin B*-RNAi-*eGFP* groups. pIZ-e*GFP* group was used as negative control. (** $p$<0.01)

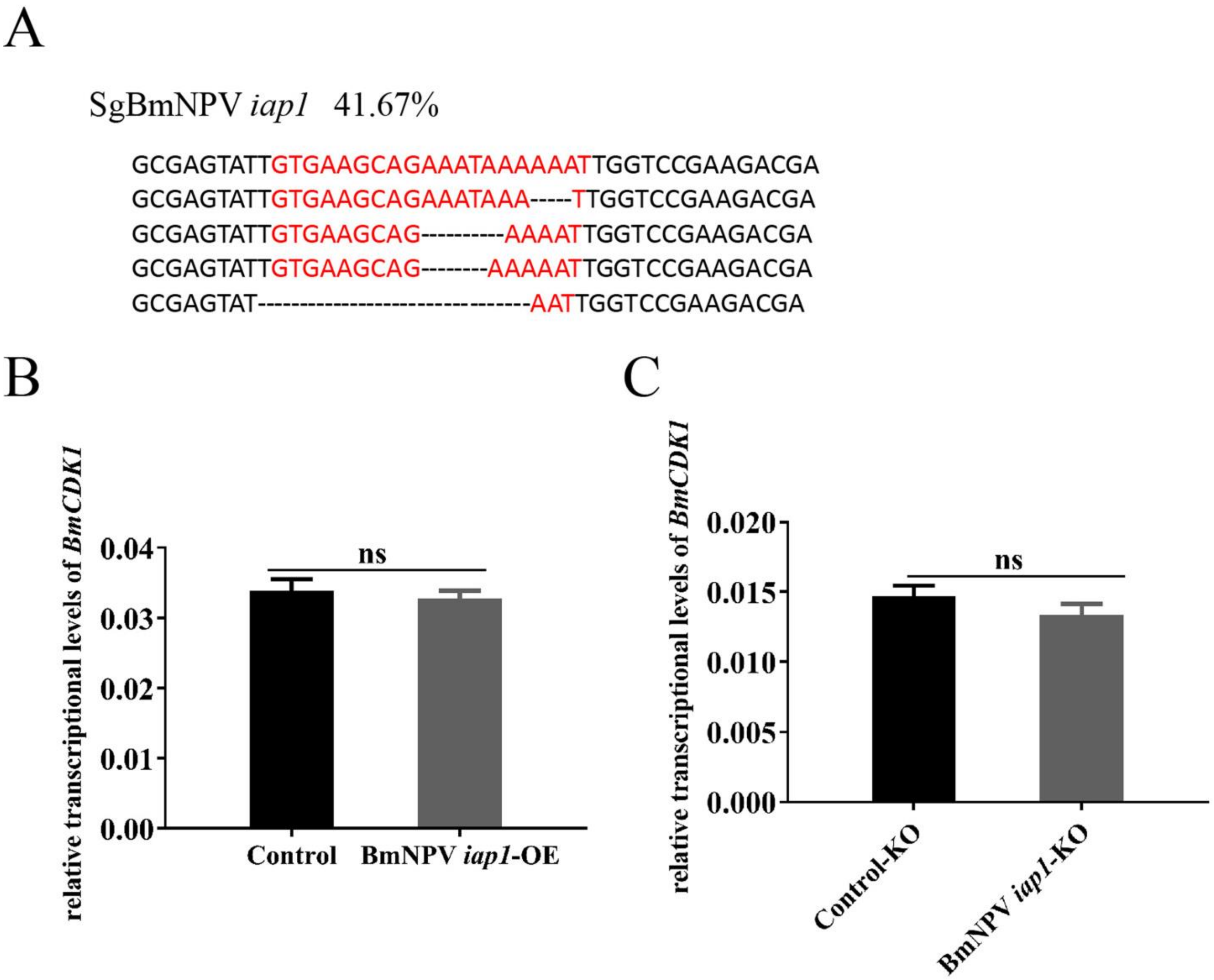


**Figure.S3 BmNPV *iap1* has no effect on cellular DNA replication and levels of *BmCDK1* mRNA.** (A) BmNPV *iap1* was edited through the CRISPR/Cas9 gene editing system. Briefly, BmNPV *iap1* was amplified by PCR, and then the PCR product was cloned into the pMD19-T vector. The recombinant plasmids were sequenced using M13 primers (B) Analyze the mRNA levels of *BmCDK1* after overexpressing BmNPV *iap1*. (C) Analyze the mRNA levels of *BmCDK1* after knocking down BmNPV *iap1* via the CRISPR/Cas9 gene editing system. ($^{ns}p \geqslant 0.05$)
